# Post-Surgery Glioma Growth Modeling from Magnetic Resonance Images for Patients with Treatment

**DOI:** 10.1038/s41598-017-01189-2

**Published:** 2017-04-27

**Authors:** Ahmed Elazab, Hongmin Bai, Yousry M. Abdulazeem, Talaat Abdelhamid, Sijie Zhou, Kelvin K. L. Wong, Qingmao Hu

**Affiliations:** 1 Research Laboratory for Medical Imaging and Digital Surgery, Shenzhen Institutes of Advanced Technology Chinese Academy of Sciences, Shenzhen, China; 2 Department of Computer Science, Faculty Computers and Information, Mansoura University, Mansoura City, Egypt; 3 Department of Computer Science, Misr Higher Institute for commerce and computers, Mansoura City, Egypt; 4 Department of Neurosurgery, Guangzhou General Hospital of Guangzhou Military Command, Guangzhou, China; 5 Department of Computer Engineering, Misr Higher Institute for Engineering and Technology, Mansoura City, Egypt; 6 Department of Physics and Mathematical Engineering, Faculty of Electronic Engineering, Menoufiya University, Al Minufiyah, Egypt; 7 School of Medicine, Western Sydney University, Campbelltown, New South Wales Australia; 8 Key Laboratory of Human-Machine Intelligence-Synergy Systems, Chinese Academy of Sciences, Shenzhen, China

## Abstract

Reaction diffusion is the most common growth modelling methodology due to its simplicity and consistency with the biological tumor growth process. However, current extensions of the reaction diffusion model lack one or more of the following: efficient inclusion of treatments’ effects, taking into account the viscoelasticity of brain tissues, and guaranteed stability of the numerical solution. We propose a new model to overcome the aforementioned drawbacks. Guided by directional information derived from diffusion tensor imaging, our model relates tissue heterogeneity with the absorption of the chemotherapy, adopts the linear-quadratic term to simulate the radiotherapy effect, employs Maxwell-Weichert model to incorporate brain viscoelasticity, and ensures the stability of the numerical solution. The performance is verified through experiments on synthetic and real MR images. Experiments on 9 MR datasets of patients with low grade gliomas undergoing surgery with different treatment regimens are carried out and validated using Jaccard score and Dice coefficient. The growth simulation accuracies of the proposed model are in ranges of [0.673 0.822] and [0.805 0.902] for Jaccard scores and Dice coefficients, respectively. The accuracies decrease up to 4% and 2.4% when ignoring treatment effects and the tensor information, while brain viscoelasticity has no significant impact on the accuracies.

## Introduction

Gliomas are a primary brain tumors that arise from the glial cells due to disruption of the normal brain cell growth. Gliomas make up approximately 30% of tumors of brain and central nervous system and 80% of all malignant brain tumors^1^. World Health Organization (WHO) divides glioma according to the degree of malignancy and other factors to four grades from I to IV^2^. Grades I and II (known as low grade glioma, LGG) tend to be less malignant and slow-growing. These tumors account for about 25% of all glioma patients who may survive for many years (3–8) and have a high quality of life during that period^3^. On the other hand, grades III and IV, known as high grade glioma (HGG), are highly malignant tumors that quickly lead to death. HGG, particularly glioblastoma multiforme, grows very fast and invades surrounding tissue. Unlike LGG, the prognosis of HGG is poor and, most likely, subject to recur after treatment with average survival time of 1 year^4^. However, LGG are vulnerable to transformation to grades III and IV after variable period of time. In a study on the transformation of LGG^5^, it was observed that 60% of the patients with LGG progressed to HGG.

Generally, glioma treatment comes in a form of surgery, radiotherapy, chemotherapy, or, most likely, a combination of them with the guidance of medical imaging techniques such as magnetic resonance imaging (MRI), computed tomography (CT) and diffusion tensor imaging (DTI). MRI is one of the most commonly used imaging modalities in diagnosis and treatment planning of gliomas. It can open a window to navigate the brain tissues and visualize the pathology that helps in identifying and tracking evolution of tumors. On the other hand, DTI can provide directional information of the fiber tracts that glioma cells preferentially migrate through.

Using magnetic resonance (MR) images, one can study the tumor growth over time from different time points using mathematical models. Such modeling can provide a better understanding of the physiology of the tumor growth, help to quantify the tumor aggressiveness, and improve therapy planning by better defining the invasion margins based on estimation of local tumor cell density^6^. However, this is not a straightforward task since tumors have different infiltration levels, complex shapes, anisotropic diffusions, and different properties of brain tissues. In addition, the high cost and the stability problems of the numerical solutions of such modeling make the operability very difficult. The growth modeling becomes even harder in presence of treatment.

Basically, tumor growth models can be divided into two categories^7^: microscopic and macroscopic models. The macroscopic models use couples of partial differential equations (PDEs) to describe the tumor growth through tumor cell proliferation and the invasion of tumor cells to the surrounding tissues. The earliest mathematical model was proposed by Tracqui^8^ using reaction diffusion (RD) model to isotropically simulate the spatio-temporal change of tumor cell concentration in two-dimensional CT images. Tumor cells diffuse with different rates according to the surrounding tissues^9^, i.e., white matter (WM), gray matter (GM), and cerebrospinal fluid (CSF). The diffusion in WM is faster than that in GM while it stops by CSF^10^. Swanson *et al*.^11–13^ used a spatial function to represent the heterogeneity of the diffusion coefficients in WM and GM guided by tissue segmentation of an anatomical atlas. Yuan *et al*.^14^ modified the RD equation by introducing a weighted parameter to balance the diffusion coefficient of the WM and GM, and local region similarity measure using normalized Bhattacharyya distance was estimated to determine the weighted parameter guided by level set function. Recently, the model was extended to include the viscous stress tensor^15^. Similarly, we previously proposed content based modified RD model using a weighted parameter that measures the WM proportion in a small window^16^.

DTI was employed to guide the simulation of the anisotropic nature of glioma cell diffusion^6, 7, 17–21^. Jbabdi *et al*.^17^ proposed one of the earliest models of anisotropic growth using DTI and showed that it could better predict the spiky nature of tumor shapes. Clatz *et al*.^6^ used DTI to assign anisotropic diffusion in WM. Because of high anisotropy in most parts of WM, the previous two approaches led to diffusivities that are much lower than gray diffusion in the directions orthogonal to the fibers. Moreover, the high ratios of anisotropy encountered in those two models are computationally expensive. Therefore, Rekik *et al*.^18^ proposed WM tumor diffusion tensor that can handle these drawbacks using an anisotropic Eikonal equation introduced by Konukoglu *et al*.^7^ to describe the time at which the evolving tumor front passes through a specific location. Similar to Konukoglu *et al*.^7^, Mosayebi *et al*.^20^ computed tumor invasion based on the geodesic distance obtained from DTI information. Painter and Hillen^21^ developed a mesoscopic model for glioma invasion based on the individual migration pathways of invading cells along the WM tracts.

To consider the tumor mass effect, Clatz *et al*.^6^ used biomechanics in the RD model to simulate the deformation of brain structures caused by tumor development. Hogea *et al*.^22^ introduced an advection term to simulate the elastic deformation of brain tissues. In our previous work^23^ we used an enhanced Voigt model to study midline shift induced by tumor growth.

The aforementioned models did not include the effect of treatment in the form of chemotherapy and/or radiotherapy. Radiotherapy is a common therapy used to control tumor cells either by killing or damaging their proliferation and is carried out after surgery in different fractionation regimen according to many factors^24^. Linear quadratic (LQ)^25^ model is the most widely used methodology to determine the effect of radiotherapy doses by estimating the probability of cell surviving due to dose of radiation. The LQ model has been used with tumor growth model^26–31^. Rockne *et al*.^26, 31^ embedded the LQ model into RD model to predict and quantify the efficacy of radiotherapy with response to various therapy schedules and dose distributions. Later, Corwin *et al*.^28^ extended this work and investigated generating patient-specific and biologically-guided radiotherapy dose plans. Roniotis *et al*.^27^ included the radiotherapy effect in the RD equation using LQ model guided by DTI information extracted from atlas.

The other treatment regimen is to use chemotherapy. Chemotherapy acts on rapidly proliferating cells by interfering with the cell-cycle and other cell-cycle specific targets. Swanson *et al*.^11^ introduced a simple technique to incorporate homogenous and heterogeneous drug delivery of chemotherapy into tumor growth model. The loss term due to chemotherapy can be embedded into the RD model as a proportion of tumor growth rate^32^. Powathil *et al*.^33^ used a log-kill model to represent the cell death caused by the chemotherapy in the RD model.

Although progresses have been made in tumor growth modeling, most of the current models focus on pre-surgery MR images^6, 7, 12, 14–17, 26–28, 30, 31, 33^. Because most glioma patients likely undergo surgery, studying tumor growth modeling after surgery is of great importance. In addition, majority of current models focus only on one treatment regimen^11, 27, 28, 31^ which make these models, clinically, less effective. Even though some models included the effect of different treatment regimens, they did not efficiently consider the heterogeneity of brain tissue^11, 33^ and viscoelasticity of the brain. Furthermore, the stability of these models are not guaranteed in real application and may be costly due to long time simulation.

Tumors are subject to recur in many cases because some tumor cells can be incidentally missed or deliberately left if the tumor bulk resection has risky consequences. Therefore, modeling tumor growth after surgery is of great importance to assist the prognosis and the future treatment by providing directional and quantitative information. To this end, we propose a new RD model that accounts for both radiotherapy and chemotherapy effects to model the post-surgery growth of glioma from MR images and to adopt the heterogeneity of brain tissues. In addition, our model considers the viscoelasticity of brain tissues using Maxwell-Weichert model and ensures the stability of the numerical solution as well as low computational cost.

## Material and Methods

### MR datasets

In this study, 9 LGG patients were recruited who underwent surgery followed by, if any, chemotherapy and/or radiotherapy from Guangzhou General Hospital of Guangzhou Military Command, Guangzhou, China. All patients provided informed written consent. The study was approved by the ethics committee of the Shenzhen Institutes of Advanced Technology (Chinese Academy of Sciences) Review Board and all experiments were carried out in accordance with the approved guidelines and regulations. For each subject, there are 3–7 time-point scans available. At every time-point, MR images include T1, T1Gd, T2, FLAIR, as well as DTI, all with an in-plane matrix size of 512 × 512 pixels (0.468 mm × 0.4688 mm) and 20–25 slices with distance between slices being about 6.5 mm. The T1 Gd MR image is very important in scanning brain tumor and it is based on the disruption of the blood brain barrier and/or the abnormal vascularity that allows the accumulation of the agent within the lesion^34^. The MRI enhancement depends on the amount of the delivered contrast and this enhancement was found to be correlated with the cell proliferation in gliomas^35^. Although tumor cell density cannot be measured from MR images, it is still a possible reason for some tumor cells being left after resection.

The standard of care for patients receiving chemotherapy is 6 cycles, each has 150–200 mg/m^2^ Temozolomide given 5 days every 28 days. On the other hand, the conformal radiotherapy delivery after surgery includes totally 60 Gy (joule/kilogram) divided into 30 daily fractions. Summary of subjects’ information under study is given in Table 1.

**Table 1:** Summary of the 9 patients’ data included in this study.

Patient	Age	Sex	Diagnosis	Location	Treatment regimen	Time (days)
1	37	F	Diffuse Astrocytoma (WHO II)	L temporal lobe	Chemotherapy + Radiotherapy	81
2	33	M	Oligodendrogliomas (WHO II)	R frontal lobe	Chemotherapy only	123
3	40	M	Dysembryoplastic neuroepithelial tumor	R frontal lobe	—	139
4	27	M	Small cell Astrocytoma (WHO II)	L frontal lobe	—	243
5	40	M	Astrocytoma (WHO I-II)	L frontal lobe	Chemotherapy only	329
6	41	M	Oligodendrogliomas (WHO II)	R frontal lobe	—	412
7	31	M	Ganglioglioma (WHO I-II)	L frontal lobe	—	686
8	50	M	Small cell Astrocytoma (WHO II)	R frontal lobe	Chemotherapy only	252
9	22	M	Small cell Astrocytoma (WHO II)	L frontal lobe	Chemotherapy + Radiotherapy	305

### Image preprocessing

**Figure 1:**
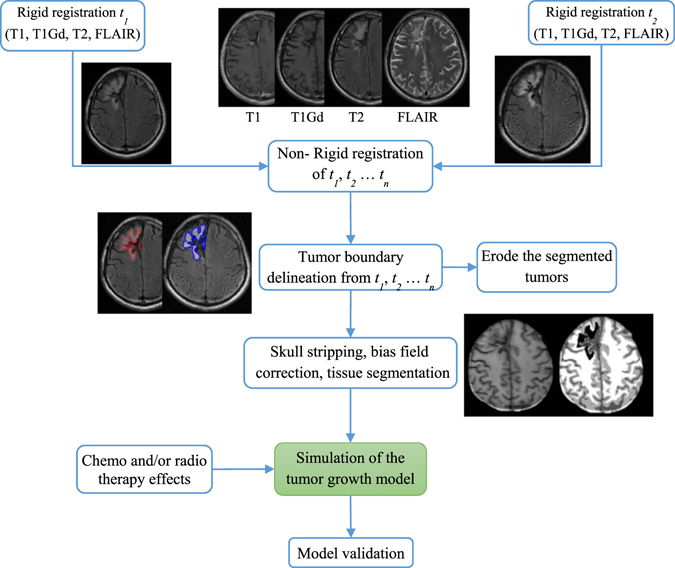
The processing pipeline of the proposed glioma growth model.

Preprocessing is of particular importance and has to be done carefully otherwise the performance of the model will be inaccurate, particularly, the MR images were scanned after surgery. The initial step is registration to align all MR images to a common space. This is done by rigidly registering all the MR modalities of same time together, e.g. *t*_*1*_, followed by non-rigid registration for all other available time-points *t*_*i*_ scans. For both rigid and non-rigid registration, we use the publically available software 3D Slicer^36^. Afterwards, tumor boundaries are manually segmented guided by an expert in this field (HB) using an in-house software. The next step is to peel the skull, i.e. separate the skull from the brain, to act as boundary condition that prevents the tumor to grow outside the brain. Generally, skull stripping can be done automatically using some commonly used software e.g. 3D slicer^36^. However, in some MR images, the cavity is close or next to the skull which makes the CSF undistinguishable by the skull stripping software. Therefore, we tackle this challenge by manually delineating the skull. Then, grayscale inhomogeneity resulted from bias field of the MRI scanner is corrected before brain tissue segmentation to avoid misclassification of WM, GM, and CSF. In this work, the grayscale inhomogeneity is corrected using the method in^37^ while brain tissue segmentation is performed by employing our algorithms in refs 37 and 38. Figure 1 shows the preprocessing steps of our model.

### Mathematical background on RD model

The biological growth process of tumor can be modeled using a semi-linear parabolic PDE known as RD model. Basically, the RD model represents the rate of change of tumor cell density by two terms (Equation 1): diffusion (motility) of tumor cells and the net proliferation of tumor cells^12^. The original RD model is defined as follow:

1 $$\frac{\partial u(x,t)}{\partial t}=\nabla \cdot (D\nabla u(x,t))+f(u(x,t))$$

where *u*(*x, t*) is the tumor cell density in position *x* at time *t*, *D* is the diffusion coefficient, and ∇ is the gradient operator. In this work we consider *f*(*u*(*x, t*)) to be logistic function which is defined as follow:

2 $$f(u(x,t))=\rho u(x,t)(1-u(x,t))$$

where *ρ* represents the proliferation rate.

Constant value of *D* yields isotropic diffusion and consequently the tumor will grow isotropically which is not precise to describe the glioma growth. Alternatively, *D*(*x*) can be used to represent the heterogeneity of the brain tissues WM, GM, and CSF. For anisotropic growth, diffusion tensor $\bar{{\boldsymbol{D}}}(x)$ extracted from DTI can give directional information of the preferred glioma growth. The RD model is completed by a no-flux boundary condition as barrier to stop the growth beyond the brain boundary using:

3 $$D(x)\nabla u\cdot {n}_{\partial {\rm{\Omega }}}=0$$

where *n*_*∂Ω*_ is the normal vector at the domain boundary surface *∂Ω*.

### Proposed growth model

We modify the original RD model in Eq. (1) by proposing PDE to include the effects of viscoelasticity of brain, chemotherapy, and/or radiotherapy. The proposed RD model is:

4 $$\frac{\,\partial u(x,t)}{\partial t}=\nabla \cdot (\bar{{\boldsymbol{D}}}(x)\nabla u(x,t))+\nabla \cdot (\hat{D}\nabla \sigma )+f(u(x,t))-R(u(x,t))-C(u(x,t))$$

where *σ* in the second term represents the normal stress in the brain tissue and $\hat{D}$ is the stress diffusion tensors represented by diagonal tensor with negative values^39^, while *R*(*u*(*x, t*)) and *C*(*u*(*x, t*)) are, respectively, the loss terms due to the radiotherapy and chemotherapy. For the proliferation term, *f*(*u*(*x, t*)), tumor cells mitosis and necrosis are commonly assumed to grow exponentially^7, 8, 12, 26, 31^ which makes the logistic growth (Equation 2) more accurate on the time scale considered^12^. A more detailed information for the other terms of Eq. (4) are given below.

### Viscoelastic model

**Figure 2:**
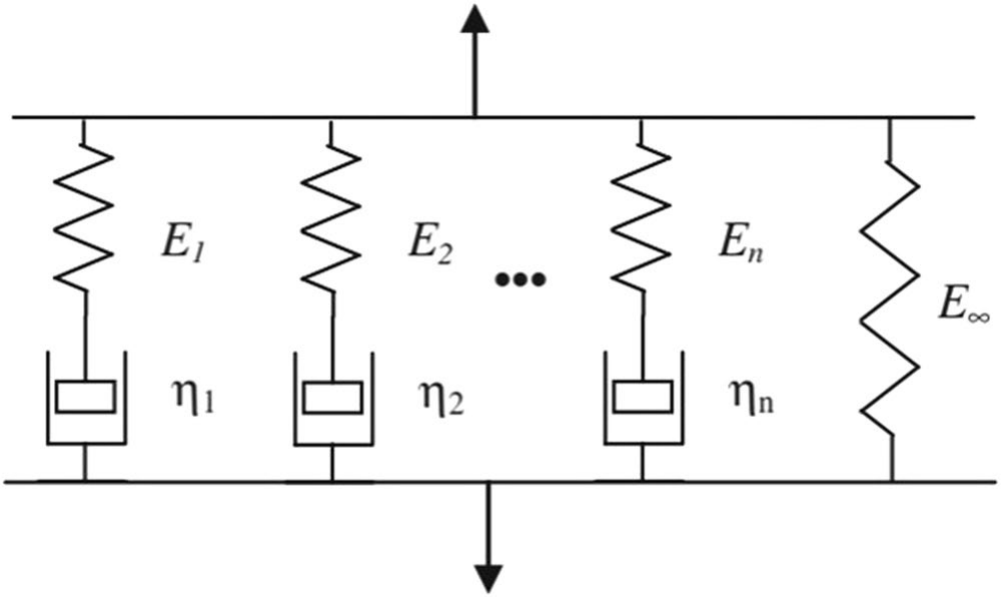
Schematic view of Maxwell-Weichert model of *n* Maxwell elements in parallel and free spring.

The brain can be considered as a medium with viscoelastic behavior^40^. In literature, there are many models to describe such behavior^41^. We choose the generalized Maxwell model (hereinafter, Maxwell-Weichert) as it can simulate the deformation and relaxation behavior of the viscoelastic material. The Maxwell-Weichert model combines several Maxwell elements assembled in parallel^42^. Each Maxwell element consists of pure elastic spring and viscous dashpot connected in series. Schematic view of the Maxwell-Weichert model is illustrated in Fig. 2.

The relaxation modulus of the model in Fig. 2 is then represented by:

5 $$E(t)=\sum _{k=1}^{n}{E}_{k}{e}^{\frac{-t}{{\tau }_{k}}}+{E}_{\infty }$$

where *E*_*k*_ is the Young’s modulus of spring *k*, ${\tau }_{k}=\frac{{\eta }_{k}}{{E}_{k}}$ stands for the relaxation time of each Maxwell element, $\,{\eta }_{k}$ is the viscosity coefficient, and *E*_∞_ represents the Young’s modulus of the free spring. Using Boltzmann integral^41, 42^, the constitutive relationship between stress and strain can be defined by:

6 $$\sigma (t)=(\sum _{k=1}^{n}{E}_{k})\nabla \varepsilon (t)+\,{\int }_{0}^{t}(\sum _{k=1}^{n}\frac{{E}_{k}}{{\tau }_{k}}{e}^{-\frac{t-s}{{\tau }_{k}}})\nabla \varepsilon (s)ds\,$$

where *ε* stands for the strain.

### Radiotherapy effect

The LQ model^25^ is the most common methodology to determine the effect of radiotherapy doses by estimating the probability of cell surviving *S* due to dose of radiation using:

7 $$S={e}^{-\alpha {d}_{i}(x,t)-\beta {d}_{i}{(x,t)}^{2}}$$

where *d*_*i*_(*x, t*) is the radiation dose and *α* (units Gy^−1^) and *β* (units Gy^−2^) are, respectively, the linear and quadratic radiobiology coefficients that represent the tissue response. The tumor cell loss is then calculated by:

8 $$r(x,t,{d}_{i})=\{\begin{array}{ll}0, & no\,\,radiotherapy\\ 1-S[\alpha ,\beta ,{d}_{i}(x,t)] & during\,\,radiotherapy\end{array}$$

Using Eq. (8), the radiotherapy effect term *R*(*u*(*x, t*)) in Eq. (4) can be calculated using:

9 $$R(u(x,t))=r(x,t,{d}_{i})\cdot u(1-u)$$

### Chemotherapy effect

Chemotherapy is used to stop or slow down tumor growth either by administrating it before, during, or after radiotherapy. Chemotherapy is commonly hypothesized to damage tumor cells which are proportional to the growth rate^33^. However, this is not precise since the chemotherapy is delivered to the whole body, unlike radiotherapy, and the absorption of drug by tissues can differ accordingly. To overcome this shortcoming, we propose to relate the heterogeneity of tissues with the absorption of the chemotherapy. Considering the loss due to chemotherapy in Eq. (4) is assumed to be:

10 $$C(u(x,t))=k(x,t)u(x,t)$$

where (*k*(*x, t*) is the cell death rate due to chemotherapy^32, 33, 43^. Since tumor grows faster in WM than in GM and tumor cell density in GM is higher than that in WM, we propose replacing *k*(*x, t*) in Eq. (10) with the following formula to adopt heterogeneity of tissue absorption of the drug:

11 $$\bar{k}(x,t)=\{\begin{array}{cc} & \{\begin{array}{c}k(x,t)x\in GM\\ \frac{k(x,t)}{k(x,t)+\omega }x\in WM\end{array}\\ 0, & no\,chemotherapy\end{array}$$

where *ω* is a parameter to be the proportion of WM tissue within a small local window^16^.

### Numerical solution

By combining the growth model components together, Eq. (4) can be rewritten using the following spatio-temporal integro-differential equation:

12 $$\frac{\partial u(x,t)}{\partial t}=\nabla \cdot ({\theta }_{1})+\nabla \cdot (\hat{D}\nabla {\theta }_{2})+u(x,t)[(\rho -r(x,t,{d}_{i})).(1-u(x,t))-\bar{k}(x,t)]$$

where *θ*_1_ and *θ*_2_ are, respectively, defined below:

13 $${\theta }_{1}=\bar{{\boldsymbol{D}}}(x)\nabla u(x,t)$$

14 $${\theta }_{2}=(\sum _{k=1}^{n}{E}_{k}+{\int }_{0}^{t}\sum _{k=1}^{n}\frac{{E}_{k}}{{\tau }_{k}}{e}^{-\frac{t-s}{{\tau }_{k}}}ds)\nabla \varepsilon (s)$$

In Eq. (13), the diffusion tensor $\bar{{\boldsymbol{D}}}(x)$ is represented by a 3 × 3 positive symmetric matrix extracted from DTI to provide directional information of the preferred anisotropic tumor growth. For simplicity and computation speed-up, $\bar{{\boldsymbol{D}}}(x)$ can be constructed using^18^:

15 $$\bar{{\boldsymbol{D}}}(x)=E(x)[diag({e}_{1}(x){D}_{WM},{D}_{GM},{D}_{GM})]{E}^{T}(x)$$

where *D*_*WM*_ and *D*_*GM*_ represent the diffusion coefficients for WM and GM, respectively, and *E*(*x*) is a matrix of sorted eigenvectors of *DTI*(*x*) while *e*_1_(*x*) is the normalized largest eigenvalue of *DTI*(*x*)^18^.

Solving Eq. (12) with the boundary condition given in Eq. (3) has to be done very carefully as the stability of the numerical method may affect the performance and the existence of the numerical solution. Generally, there are 2 common numerical techniques to solve the RD model of tumor growth: finite elements and finite differences. For the proposed model, we choose the finite differences method because of its easiness and convenience to the nature of the digital image representation. However, the numerical solution of the proposed RD model (Eq. 12) is complex with conditional stability, especially when DTI information is employed. To ensure the numerical stability of the proposed model, we follow the discretization method proposed by Weickert^44^ and its extended form by Mosayebi *et al*.^45^, which employed first order finite differences and directional discretization. In our case, the mesh size is set typical to the MR image size which is represented by a Cartesian coordinate system on a cubic grid. In addition, we consider the Neumann boundary condition by setting the tumor cell concentration on the brain boundary to zero.

### Model assumptions

In our model, we assume that the effect of the treatment terms is always less than the proliferation term. This assumption is logically sound as the gross tumor volume is still increasing (see the Figures of clinical MR images experiments). In addition, we assume that effects of both radiotherapy and chemotherapy are independent with no interference between them. In fact, it is not clear whether the major benefit comes from either concomitant administration of chemotherapy with radiotherapy, or from the six cycles of adjuvant chemotherapy, or both^30^. Finally we assume that, both radiotherapy and chemotherapy have no effect on the cavity.

### Experiments

We carried out experiments on both synthetic and clinical MR images to evaluate the performance of the proposed method. Unless mentioned otherwise, the main parameters and the associated values from references are summarized in Table 2. In our experiments we set *n* = 1 which proved to be sufficient since the mass effect of the MR images for LLG subjects are less serious than that for HGG subjects. Subsequently, there are two Young’s moduli *E*_1_ and *E*_∞_: the first for the Maxwell element while the latter for the free spring. For the LQ model parameters, the radiobiological ratio (α/β) was chosen to be 10 Gy as done in refs 26, 28 and 31.

**Table 2:** The description of the model parameters used in the experiment of the MR images.

Parameter	Description	Value
*n*	Number of Maxwell elements	1
***D***_***GM***_	Diffusion coefficient in GM	0.0013 (cm^2^/day)^11, 12, 14, 16^
***D***_***WM***_	Diffusion coefficient in WM	5*D*_*GM*_^11, 12, 14, 16^
***ρ***	Proliferation rate	0.012 (day^−1^)^12, 49^
***E***_**1**_	Young’s modulus of Maxwell element	3156 Pa^50^
***E***_∞_	Young’s modulus of the free spring	*E*_1_ = 6*E*_∞_^50^
***η***_**1**_	Viscosity of the Maxwell element	8.9×10^−8^ Pa. Sec^50^
***α***/***β***	LQ radiobiological ratio	10 Gy^26, 28, 31^
***k***(***x, t*** **)**	Chemotherapy loss	0.0196 (day^−1^)^32, 33, 43^
$\hat{{\boldsymbol{D}}}$	Stress diffusion tensors	−10^−14^ cells/Pa day^51, 52^

To evaluate the accuracy of the model, we compared the simulated growth (*S*) with the ground truth (*GT*) of the corresponding scan using two different evaluation criteria: *Jaccard* score (*JS*)^46^ and *Dice* coefficient (*DC*)^47^ that are defined as:

16 $$JS=\frac{|S\cap GT|}{|S\cup GT|}$$

17 $$DC=\frac{2\,|S\cap GT|}{|S|+|\,GT|}$$

These two criteria are used to measure the degree of overlapping between *GT* and *S* and their values are in the range of 0 and 1. The high values of *JS* and *DC* correspond to accurate simulated growth.

### Synthetic tumor growth

**Figure 3:**
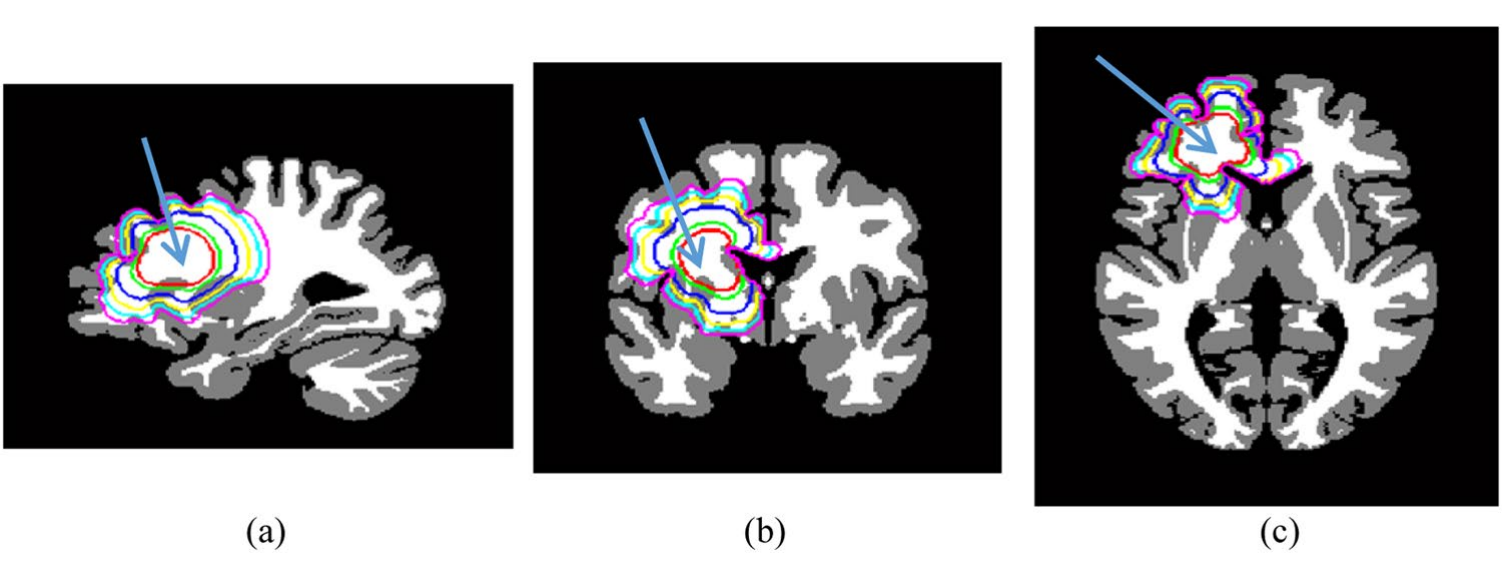
Simulation of the synthetic growth of tumor from single point on MNI atlas. The red, blue, and cyan contours represent the tumor boundaries for 1, 1.5, and 2 years, respectively, with treatment effect; while the green, yellow, and magenta contours represent the tumor boundaries for 1, 1.5, and 2 years, respectively, without treatment effect. (**a**) Sagittal slice, (**b**) coronal slice, and (**c**) axial slice.

We first utilized the corrected MNI atlas^48^ to simulate the tumor growth from single point with and without the effect of treatment. The results shown in Fig. 3 from single point indicated by the arrow were simulated using ${D}_{GM}=0.02\,m{m}^{2}/day$ and *ρ *= 0.02 day^−1^.

**Figure 4:**
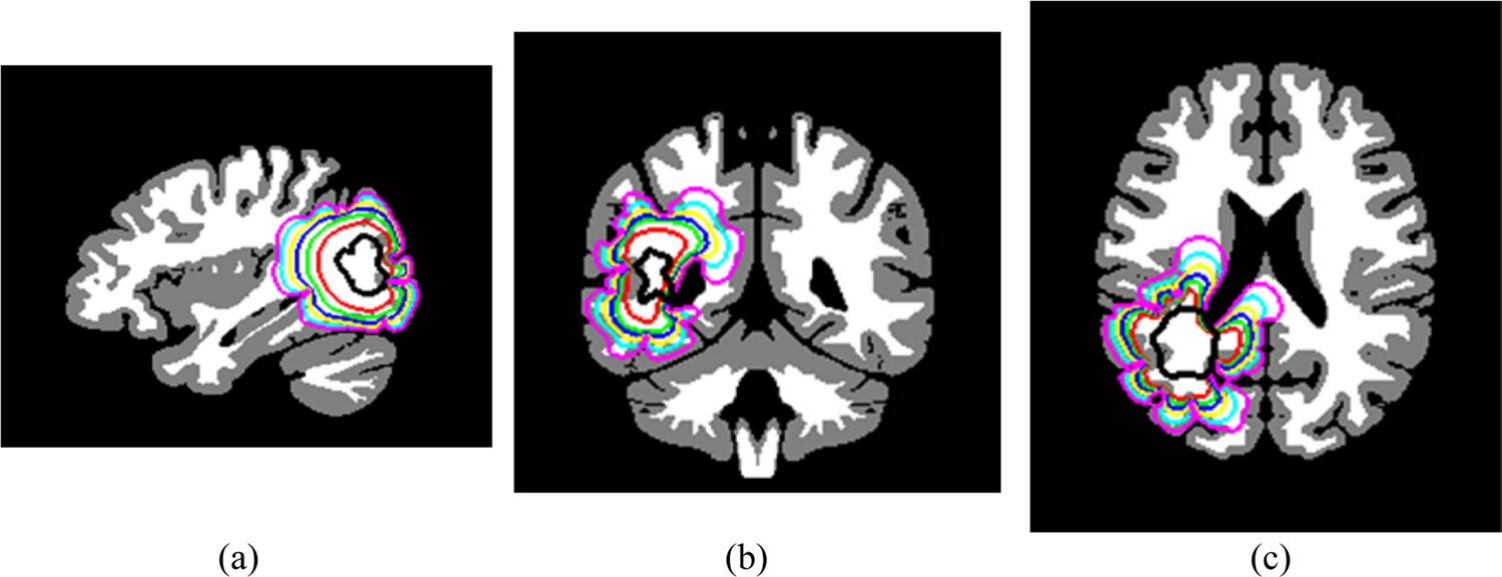
Simulation of the synthetic growth of real LGG seeded into the MNI atlas represented in the dark black color. The red, blue, and cyan contours represent the tumor boundaries for 1, 1.5, and 2 years, respectively, with treatment effect; while the green, yellow, and magenta contours represent the tumor boundaries for 1, 1.5, and 2 years, respectively, without treatment effect. (**a**) Sagittal slice, (**b**) coronal slice, and (**c**) axial slice.

The second synthetic experiment simulated the growth of real LGG seeded into the MNI atlas using the same parameters. The simulated growth with and without treatment effects was shown in Fig. 4. The treatment in the synthetic experiments followed the same standards given in the Experiments Section.

### Experiments on clinical MR images

**Figure 5:**
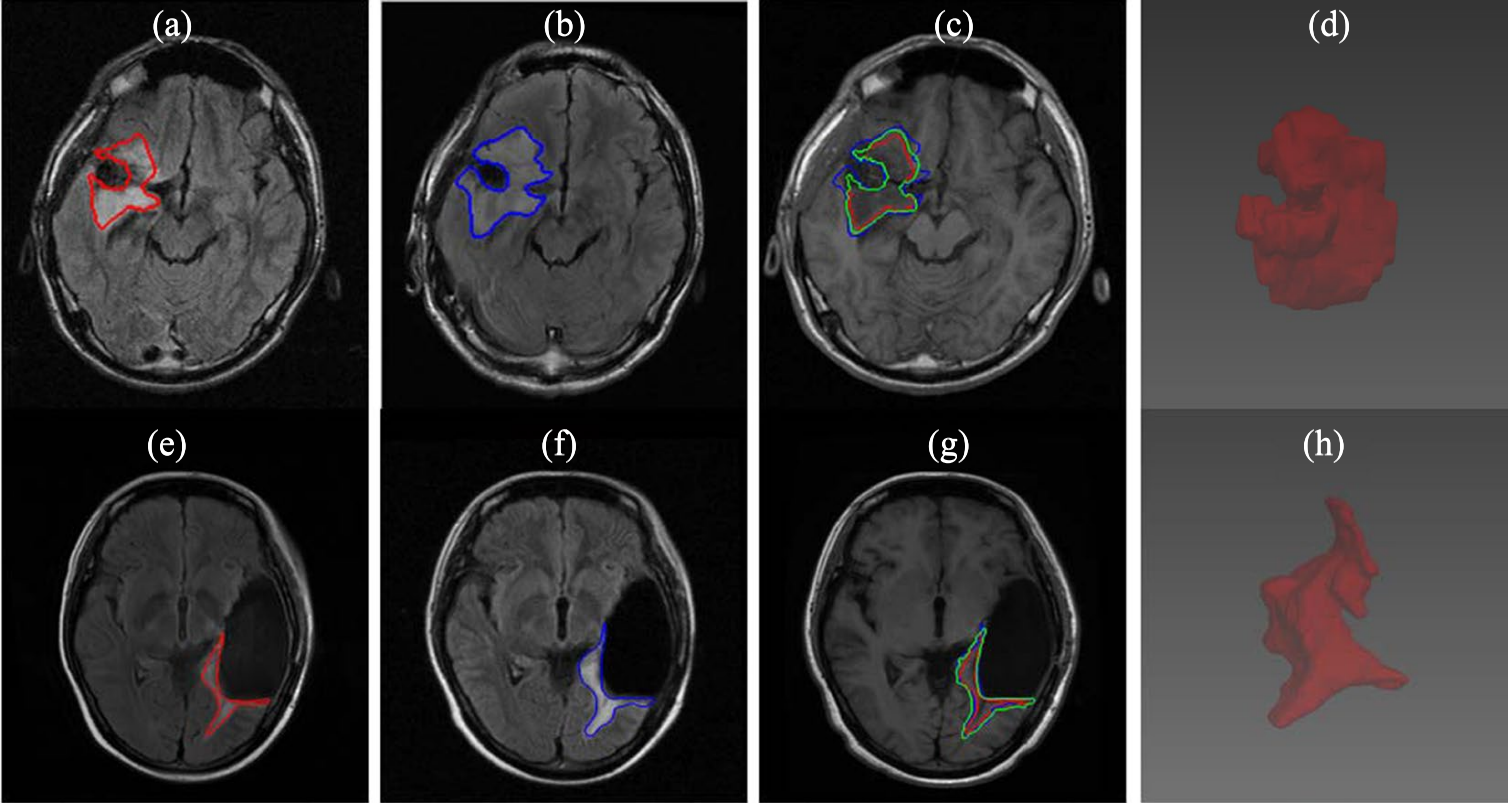
Simulation results of tumor growth from 2 LGG patients who underwent surgery followed by chemotherapy and radiotherapy. The red (**a**,**e**) and blue (**b**,**f**) contours represent the ground truths of tumor boundaries on2 serial T2 MR images while the green contours (**c**,**g**) are the simulated growths on T1 MR images. (**d**) and (**h)** are constructed 3D views of the simulated tumor growth.

**Figure 6:**
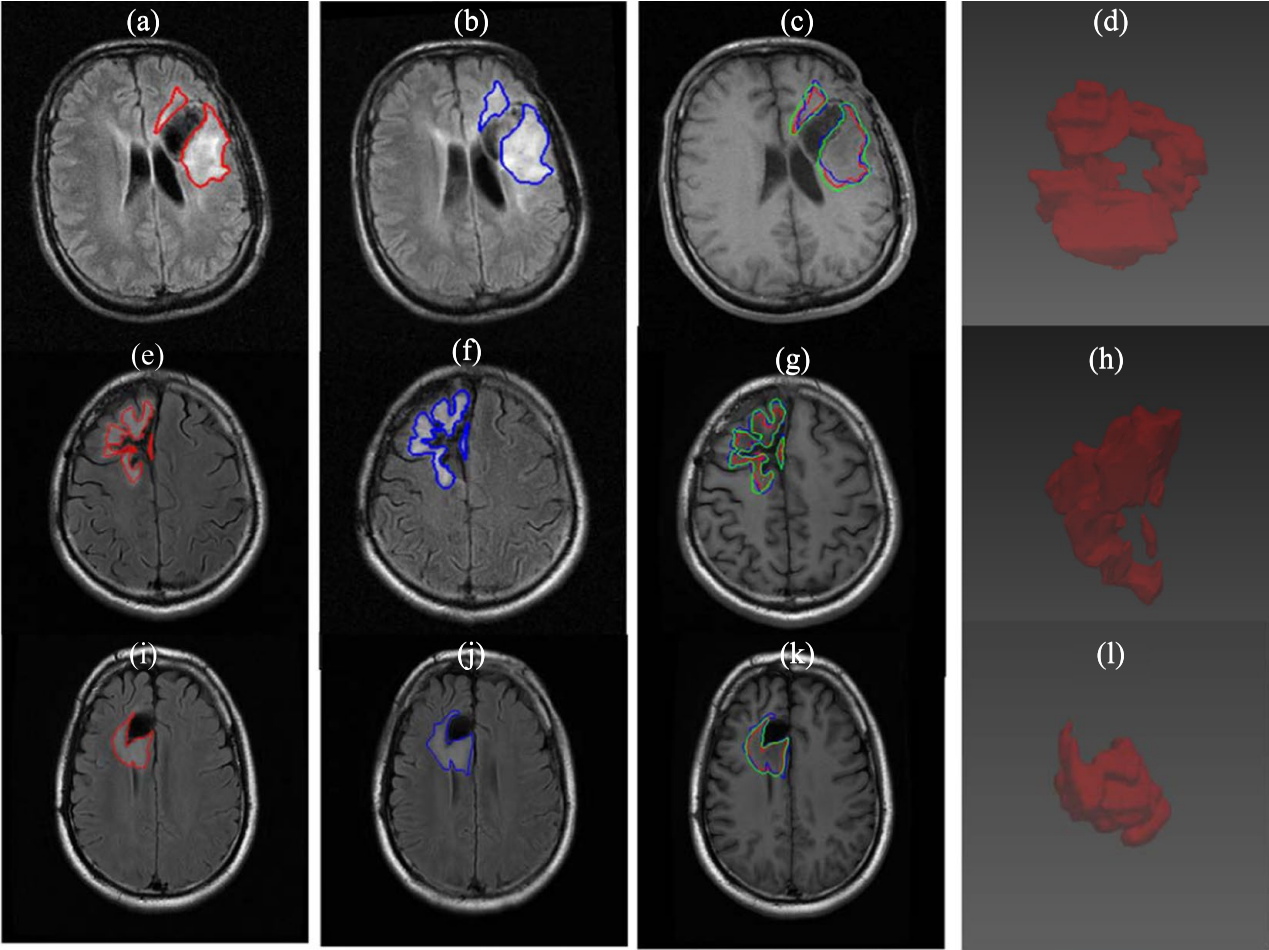
Simulation results of tumor growth from 3 LGG patients who underwent surgery followed by chemotherapy. The red (**a**,**e**,**i**) and blue (**b**,**f**,**j**) contours represent the ground truths of tumor boundaries on2 serial T2 MR images while the green contours (**c**,**g**,**k**) are the simulated growths on T1 MR images. (**d**), (**h**), and (**l**) are constructed 3D views of the simulated tumor growth.

**Figure 7:**
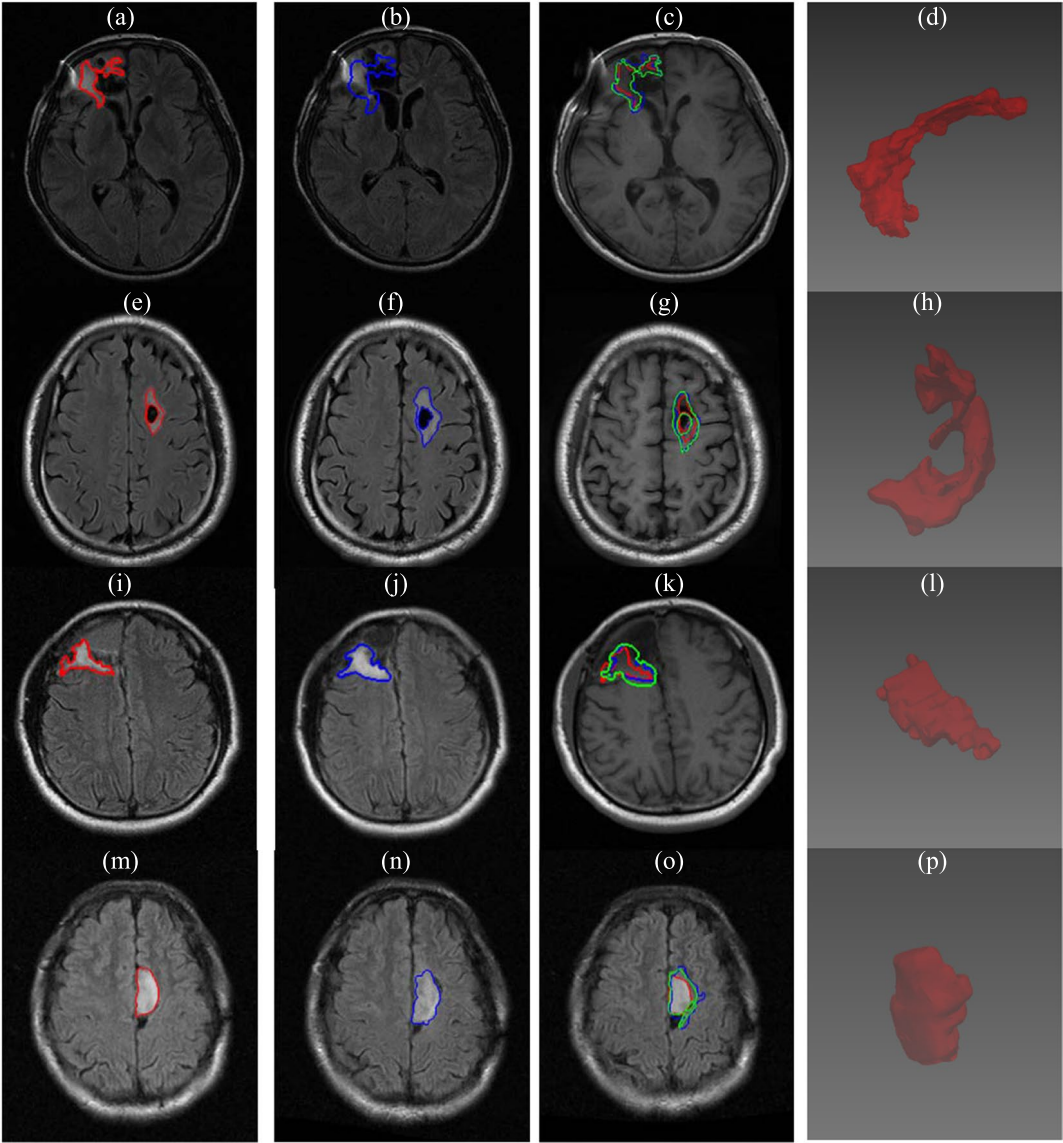
Simulation results of tumor growth from 4 LGG patients who underwent surgery without further treatment. The red (**a**,**e**,**i**,**m**) and blue (**b**,**f**,**j**,**n**) contours represent the ground truths of tumor boundaries on2 serial T2 MR images while the green contours (**c**,**g**,**k**,**o**) are the simulated growths on T1 MR images. (**d**), (**h**), (**l**), and (**p**) are constructed 3D views of the simulated tumor growth.

Experiments on the clinical MR images were carried out on 9 LGG patients who underwent surgery. The 9 MR datasets were classified as follows: 2 subjects received both radiotherapy and chemotherapy, 3 subjects received only chemotherapy, while the other 4 subjects were left without treatment. The experimental results for the above 3 groups and constructed three-dimensional (3D) views of the simulated tumor growths were shown, respectively, in Figs 5, 6 and 7. For the experiments on the clinical MR images, we took the manually delineated tumors from the first (red contours) and second scans (blue contours) as, respectively, the initial conditions and ground truths. For the detection threshold, there is no consensus in the published literature on an exact value. In addition, the detection threshold is dependent on the imaging modality and thus has to be changed accordingly. For instance, Swanson *et al*.^13^ set the detection threshold to be 0.16 for T2 image and 0.8 for T1 Gd image. To the best of our knowledge, there is no study investigates the optimal threshold. Therefore, in our case, we used *u*=0.4 as used by Tracqui *et al*.^8^ and Konukoglu *et al*.^7^ to identify the visible contour of the simulated growths (green contours).

**Figure 8:**
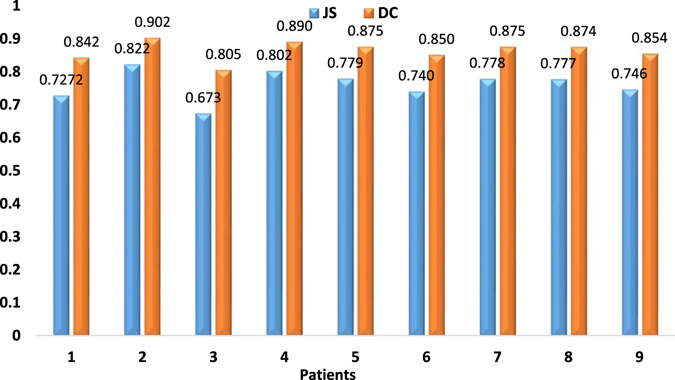
Evaluation results of the proposed method on the 9 LGG patients under study using JS and DC.

The accuracies of the proposed method on the 9 LGG patients using JS and DC were shown in Fig. 8.

### Additional experiments

To check the effects of including therapies on the growth simulation accuracy, we studied variants of the proposed method. For the two patients who received radiotherapy and chemotherapy (Fig. 5), we evaluated the role of ignoring one or two treatments on the growth simulation accuracies (Table 3). Similarly, for the 3 patients who only received chemotherapy (Fig. 6), the results when chemotherapy was ignored are summarized in Table 4.

**Table 3:** Evaluation results of the proposed method on the 2 patients with both treatments by ignoring one or two treatments.

Patient	Chemotherapy only		Radiotherapy only		No treatment	
	**JS**	**DC**	**JS**	**DC**	**JS**	**DC**
**1**	0.702	0.825	0.683	0.812	0.664	0.798
**9**	0.713	0.832	0.694	0.819	0.658	0.794

**Table 4:** Evaluation results of the proposed method on the 3 patients with chemotherapy without considering the treatment.

Patient	2	5	8
JS	0.791	0.735	0.741
DC	0.883	0.847	0.851

We also evaluated the effects of the DTI information on the growth simulation accuracies when replacing $\overline{\,D}(x)$ by *D*(*x*). Growth simulation results of all patients without including the DTI information are summarized in Table 5.

**Table 5:** Evaluation results of the proposed method on the 9 LGG patients under study using JS and DC without including DTI.

Patient	1	2	3	4	5	6	7	8	9
JS	0.719	0.807	0.661	0.785	0.76	0.722	0.754	0.761	0.73
DC	0.837	0.893	0.796	0.880	0.864	0.839	0.860	0.864	0.844

## Discussion

Modeling tumor growth aims to studying the evolution of tumor. Such modeling is important for tumor prognosis, quantifying the response to therapy, and treatment planning. In many cases, tumor is not fully resected due to the difficulty of defining tumor boundaries or serious consequences on patient’s life after the bulk resection of tumor, especially in case of LGG as there is higher expectancy of longer survival. Therefore, studying tumor growth becomes very challenging and complicated for patients after tumor resection particularly with treatments as tumor shapes will be very hard to predict.

In this paper, we proposed a new RD model that included the effects of treatments (both radiotherapy and/or chemotherapy) and brain tissue viscoelasticity. We validated the proposed method on both synthetic and clinical brain MR images with different treatment regimens. The results of 9 LGG patients show promising performances and high accuracies.

### Preprocessing challenges

Preprocessing the MR images is of particular interest and has great influence on the performance. Because these MR images were acquired after surgery, registration becomes very difficult due to the expected brain shift and the cavity. We tackle this problem by choosing the most visibly accurate registration from different nonlinear registration methods. In addition, skull stripping is also difficult because of the open skull (Figs 5e and 7a,i) which makes skull stripping fail for some MR images, as shown in Figs 5e and 7a,i. We handled this issue by manually delineating the skull mask.

### Model parameters

The proposed method has some parameters that have important impact on the growth simulation, especially *D* and *ρ*. These two parameters are highly recommended to be patient-specific for HGG. However, in our case, the 9 datasets available are all LGG where the cell invasion and proliferation are relatively small. In addition, for LGG, there is almost consensus in literature on the *D* and *ρ* values^11, 12, 14, 15, 17, 33, 49^. On the other hand, the parameters *α* and *β* in Eqs (7) and (8) control the relative contribution of each term of the LQ model. The value of *α*/*β = *10 Gy was used by many researchers^26, 27, 31, 33^ and showed to achieve good performance. In our case, α was chosen to be 0.027 Gy as suggested in^33^ for the two patients in Fig. 5.

### Growth simulation accuracy

Experiments on synthetic images in Figs 3 and 4 show good agreement of tumor invasions to be faster in WM, slower in GM, and zero in CSF. In addition, the effects of treatments by killing tumor cells are clear with distinguishable contours (red, blue, and cyan) from those without treatments (green, magenta, and yellow). Furthermore, the experiments prove that the tumor growth stops by ventricles and cannot cross the other hemisphere except through the corpus callosum (yellow, cyan, and magenta contours in Fig. 3c and the yellow contour in Fig. 4c).

The proposed method on the clinical MR images achieves high simulation accuracies (Fig. 8) and is able to work on both multifocal tumors (Fig. 6a and e) and monofocal ones (rest of clinical MR images). For the two patients receiving radiotherapy and chemotherapy (Fig. 5), the simulated growth accuracies were 0.727, 0.746 and 0.842, 0.854 for JS and DC, respectively. The accuracies of the three patients receiving only chemotherapy (Fig. 6) were 0.791, 0.735, 0.741 and 0.883, 0.847, 0.851 for JS and DC, respectively. Finally, the accuracies of the four patients without treatment were 0.673, 0.802, 0.740, 0.778 and 0.805, 0.890, 0.851, 0.875 for JS and DC, respectively. These results are due to directional information derived from the DTI and the inclusion of tissue heterogeneity with the absorption of the chemotherapy. However, the accuracy of the patient in Fig. 7a is low, which may be due to the artifacts of skull clamp that distorted the MR image and hence the further processing.

Treatments proved to have a significant effects on the growth simulation accuracies. For the two patients who received radiotherapy and chemotherapy (Fig. 5 and Table 3), it was found that if only the chemotherapy was considered, the JS and DC were decreased by (2.5%, 3.3%) and (1.7%, 2.21%), respectively. If only the radiotherapy was considered, the JS and DC were decreased by (4.4%, 5.2%) and (3.03%, 3.52%), respectively. Finally, if both chemotherapy and radiotherapy were ignored, the JS and DC were decreased by (6.3% and 8.8%) and (4.38%, 6.08%), respectively. Similarly, when ignoring the chemotherapy effects for the three patients who received only chemotherapy (Fig. 6 and Table 4), the growth simulation accuracies were decreased by (3.1%, 4.4%, 3.6%) and (1.9%, 2.85%, 2.33%), respectively for JS and DC. These additional experiments may imply that (1) both the chemotherapy and radiotherapy have a role in tumor growth, and (2) ignoring either treatments could result in a decrease of up to 4% in JS and DC.

When the tensor information in Eq. (15) was ignored, it was found that JS and DC were decreased by ranges of [0.8%, 2.4%] and [0.55%, 1.53%], respectively (Table 5). Apparently, the inclusion of DTI did not significantly increase the accuracy as the MR images used in our experiments were for LGG patients where the tumor cell diffusion is slow. In addition, those patients underwent surgery to include only the left tumor portion as the ground truth after the major tumor bulks were resected. However, we believe that this could be useful clinically for patients who are subject to undergo multiple surgeries. More importantly, we intend to use our model for HGG where DTI has to be included.

Finally, the role of viscoelasticity was noticed to be the least significant factor on the accuracies for LGG patients to have JS and DC decreased by ranges of [0.07%, 0.27%] and [0.1%, 0.4%], respectively. This is mainly because the growth of LGG is relatively slow and edema is often negligible. However, we included the viscoelasticity so that the proposed model in Eq. (12) could be used for both LGG and HGG.

### Relation with other models

The formulation of the proposed model is very flexible and can be considered as a general framework that can be easily configured to produce the other modified versions of the RD model. This is mainly because of the formulation of the last term in Eq. (12). For instance, excluding the viscoelasticity of brain tissues, if the r (*x, t, d*_*i*_) and $\bar{k}(x,t)$ are set to 0 with *D*(*x*) replacing $\bar{D}(x)$, our model will reproduce the model of Swanson *et al*.^12^. With the same previous configuration and slight modification to the chemotherapy effect in Eq. (11), our model will behave as the other model of Swanson *et al*.^11^. On the contrary, if the DTI information is used without the effects of the treatment and viscoelasticity of brain tissues, our model will reproduce the model of Jbabdi *et al*.^17^.

On the other hand, when including the efficacy of radiotherapy only, our model will be similar to those in refs. 27, 28 and 31. Our model can be identical to^33^ for simulating the efficacy of both chemotherapy and radiotherapy if we exclude brain tissue heterogeneity and viscoelasticity from our model (Eq. 11).

Generally, our model can be widely applied to different treatment regimens through modifications of some parameters. In addition, we believe it can be applied to other HGG, e.g. glioblastoma multiforme, after modifying the DTI information to capture their anisotropic growth and customizing the *D* and *ρ* parameters.

### Limitations

Although the proposed method has some advantages, the current study is not without limitations. On one hand, comprehensive and precise comparisons were not performed. In fact, one of the biggest challenges in studying tumor growth modeling is comparing the results with recent publications. However, this is very difficult as, to the best of our knowledge, there is no public dataset that researchers can use for benchmarking. Nevertheless, rough comparison of the proposed method with most recently published study in ref. 15 shows that, the average JS achieved by the proposed method is 0.76 which is 2.2% higher than that reported in ref. 15 (JS = 0.738).

One more limitation is that, the proposed method was only tested on MR images of LGG patients. To be more effective, the performance on other MR images of HGG patients where there will be mass effect has to be investigated but, unfortunately, such MR images are rare and currently unavailable. In addition, customization of the growth model parameters (*D* and *ρ*) will be required. Therefore, we plan to handle the aforementioned limitations in our future work when such datasets are available.

## Conclusion

We propose a new RD model for tumor growth of post-surgery LGGs. Our model includes the efficacies of both chemotherapy and radiotherapy as well as the viscoelasticity of brain tissues. Our model accuracy is investigated using different experiments on both synthetic and clinical MR images of 9 LGG patients who underwent surgery and different treatment regimens with ranges of [0.673 0.822] for JS and [0.805 0.902] for DC, respectively. To the best of our knowledge, this is the first study that includes treatment effects with brain tissues heterogeneity and viscoelasticity while ensuring the stability of the numerical solution of the model.

The proposed model aims to be clinically beneficial by providing directional and quantitative information for those patients who undergo multiple surgeries and tailor therapy for them. However, this is a preliminary work and we hope by further investigations on more datasets to be applicable in the near future.
